# Hepatitis A virus seroprevalence among children and adolescents in a high-burden HIV setting in urban South Africa

**DOI:** 10.1038/s41598-022-25064-x

**Published:** 2022-11-30

**Authors:** Nicolette M. du Plessis, Ahmad Haeri Mazanderani, Nkengafac Villyen Motaze, Makhosazane Ngobese, Theunis Avenant

**Affiliations:** 1 Department of Paediatrics, Faculty of Health Sciences, Kalafong Hospital, University of Pretoria, Private Bag X396, Pretoria, 0001 South Africa; 2 Kalafong Provincial Tertiary Hospital, Pretoria, South Africa; 3 Department of Paediatrics & Child Health, Faculty of Health Sciences, University of the Witwatersrand, Johannesburg, South Africa; 4 Centre for HIV & STIs, National Institute for Communicable Diseases, National Health Laboratory Service, Johannesburg, South Africa; 5 Medicine Usage in South Africa, School of Pharmacy, Faculty of Health Sciences, North West University, Potchefstroom, South Africa; 6 Thembisa Provincial Tertiary Hospital, Pretoria, South Africa

**Keywords:** Infectious diseases, Infectious diseases

## Abstract

Hepatitis A virus (HAV) infection is one of the most important global causes of viral hepatitis. Recent reviews suggested that HAV endemicity in South Africa could shift from high to intermediate. A hospital-based HAV seroprevalence study was conducted between February 2018 and December 2019 in Pretoria, South Africa. Systematic sampling was performed on children and adolescents (1–15 years) who attended outpatient services. Participants with a known HIV status and valid HAV serology results were included. Of the 1220 participants, the median age was 7 years (IQR: 4–11), with 648 (53.11%) males and 572 (46.89%) females. Of 628 (51.48%) HIV-infected participants, most (329, 71.83%) were both immunologically and virologically controlled or had low-level viremia (74, 16.16%). Almost three-quarters (894, 73.28%) were living in formal dwellings, and just over half (688, 56.39%) had access to clean water sources inside the house. Increasing age was associated with testing HAV IgG-positive (OR 1.25; 95% CI 1.20–1.30, *p* < 0.001), with 19.8% of participants one year of age compared with 86.7% of participants 15 years of age. This study suggests that South Africa has an intermediate HAV seroprevalence, with rates < 90% by 10 years of age (68.6%). Increased age and informal dwellings are statistically associated with HAV seropositivity, while HIV status does not significantly influence HAV seropositivity.

## Introduction

Hepatitis A virus (HAV) infection is one of the most important causes of viral hepatitis, both in South Africa and worldwide^1^. Global epidemiological data estimate that 1.5 million of people are infected annually with acute HAV, with the highest HAV-associated mortality occurring in sub-Saharan Africa and Asia^2^. Age at natural infection with HAV and burden of disease are closely linked to the economic development of a country. Shifts in age-specific prevalence patterns that reflect a transition from high to intermediate endemicity are linked to improvements in socioeconomic factors. A decrease in age-seroprevalence rates and HAV infection incidence is strongly associated with socioeconomic factors such as household income and improved clean water sources within communities^3^. Countries where HAV endemicity is changing from high to intermediate have demonstrated an increase in hepatitis A infections severity, leading to more clinically severe cases and consequently in an increase in fulminant hepatic failure secondary to HAV infection^2^. Hence, large-scale hepatitis A vaccination is likely to be cost-effective in such circumstances, as supported by the World Health Organization (WHO) recommendation for HAV vaccination in populations with < 90% seropositivity by age 10 years. Integration of HAV vaccines into the national immunization schedules is recommended in the abovementioned scenario^2^.

Prior to 2005, Southern African HAV seroprevalence rates among children aged 1–4 and 5–9 years were estimated to be as high as 83 and 92%, respectively^4^. Although the true burden of hepatitis A disease in South Africa is not known at present, a seroprevalence review of laboratory data from 2005 to 2015 demonstrated that seroprevalence rates of more than 90% are only reached in adulthood within the South African public health sector, suggesting that South Africa could be in transition from high to intermediate endemicity^5^. More recent reviews performed by Patterson et al. in 2019 supported the suspicion that South Africa is most likely changing endemicity from high to intermediate^6^.

Human immunodeficiency virus (HIV) infection is one of the most serious health concerns in South Africa. The country has the highest number of people afflicted with HIV of any country and, according to the 2019 United Nations statistics, the fourth-highest adult HIV prevalence with a rate of 19%, with only Botswana (25%), Lesotho (25%) and Swaziland (27%) recording higher prevalence rates. According to Statistics South Africa’s mid-year population estimates for 2018, the total HIV prevalence rate for the country was 13.1%. The HIV prevalence rate for all adults aged 15–49 is 19.0%, with an estimated quarter of a million children < 15 years of age living with HIV in South Africa^7,8^.

The correlation between HIV and HAV infection in adults varies according to the local epidemiology^9^. In countries with high HAV endemicity, no significant difference in HAV seroprevalence was observed between HIV-positive and HIV-negative adult cohorts^10^. In contrast, HIV-positive adults usually have a higher HAV seroprevalence than their HIV-negative counterparts in more developed countries, with low HAV endemicity^11^. HAV seroprevalence among HIV-infected children and adolescents has not been described in South Africa before. Hence, we conducted a seroprevalence surveillance study in an urban setting in healthy, HIV-infected and HIV-uninfected children and adolescents to assess current HAV endemicity.

## Methods

### Study design

A hospital-based cross-sectional analytical study was carried out. A probability sampling method was adopted using systematic sampling (screening and recruiting the 5th registered patient in the subsequent clinic queue) of children aged 1–15 years attending weekday outpatient department services and clinics at Kalafong Provincial Tertiary Hospital in Pretoria, South Africa. Recruitment was performed between February 2018 and December 2019. Children and adolescents with minor illnesses (i.e. illnesses not necessitating admission) and/or those attending follow-up visits were enrolled once written parental consent and patient assent (> 8 years) were obtained. Participants and their caregivers were interviewed using a standardized questionnaire, and blood samples were collected for anti-HAV IgM and IgG testing. Anti-HAV serology testing (both IgM and IgG) was performed using the Abbott ARCHITECT i2000SR immunoassay analyzer. Only participants with a known HIV status (laboratory evidence of HIV-infected or HIV-uninfected status) and valid anti-HAV IgM and IgG results were included in the study. HIV-infected participants were recruited from the paediatric HIV clinic, a weekday clinic for HIV-infected children and adolescents. All HIV-infected children and adolescents have access to age-appropriate universal antiretroviral therapy at the clinic.

### Statistical analysis

The sample size was calculated assuming the frequency of HAV IgG to be 50%, with power set at 80% and precision at 5%. The results of these assumptions give a sample size of 383 per age group: 1–5 yrs; 6–10 yrs; 11–15 yrs. The total sample size of the study was calculated to be 1149.

The percentages of Hepatitis A IgG- and IgM-positive results, as well as variables associated with these results, are reported as proportions of study participants. Participant age was reported as the median with interquartile range (IQR) in years. A multivariable logistic regression analysis was performed, during which odds ratios (ORs) and 95% confidence intervals (CIs) were calculated to determine whether specific participant characteristics were associated with positive hepatitis A IgG serostatus. Ten variables were included in the model, namely, age of the participant (as a numerical variable), HIV status of participant, maternal HIV status during pregnancy with participant, participant residence (informal dwelling or not), participant access to running tap water inside place of residence, pit latrine ablution facilities at place of residence, employment status of primary caregiver, participant attendance at day-care and/or school, caregiver receipt of a government support grant, and caregiver level of education. Inclusion in the multivariable model was dependent on an associated univariable logistic regression *p* value of < 0.2. Statistical analysis was performed using R version 3.6.3.

### Ethical and legal considerations

All methods were carried out in accordance with relevant guidelines and regulations. The research protocol was approved by the University of Pretoria Medical Research Ethics Committee 362/2017. Informed consent and assent (where applicable) were obtained from all subjects and/or their legal guardian(s). All patient data and laboratory results were anonymised, kept confidential and stored in a password-protected database.

## Results

**Figure 1:**
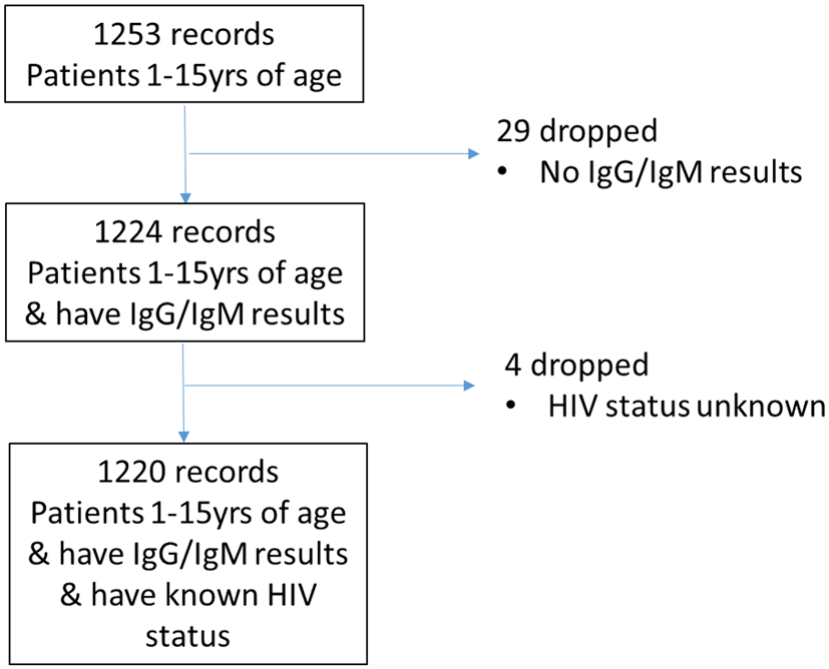
Diagram indicating participant inclusion.

Among 1253 participants between the ages of 1 and 15 years enrolled in the study, 1220 were included in the final analysis (Fig. 1).

The median age of the enrolled participants was 7 years (IQR: 4–11), with 648 (53.11%) males and 572 (46.89%) females. There were a total of 628 (51.48%) participants who were known to be HIV-infected and receiving antiretroviral therapy. Only one (0.08%) participant indicated that they had previously received an HAV vaccine, and 5 (0.41%) had a known previous HAV infection. The majority of participants had access to a flush toilet, with 22.05% using a pit latrine. Although almost three-quarters (894, 73.28%) of households were living in formal dwellings, just over half (688, 56.39%) had access to clean water sources (taps) inside the house. A summary of variables, including indicators of socioeconomic status, is presented in Table 1.

**Table 1:** Study participant variables, including socioeconomic status indicators.

Variable	N	Yes, n (%)	No, n (%)	Missing, n
Previous HAV diagnosis	1211	5 (0.41)	1 206 (99.1)	9
Prior HAV vaccination	1219	1 (0.08)	1 218 (99.92)	1
HIV-infected	1220	628 (51.48)	592 (48.52)	–
Maternal HIV infection during pregnancy	1196	617 (51.59)	579 (48.41)	24
Resides in an informal dwelling	1220	326 (26.72)	894 (73.28)	–
Inside water tap	1220	688 (56.39)	532 (43.61)	–
Pit latrine	1220	269 (22.05)	951 (77.95)	–
Caregiver without schooling	1220	20 (1.64)	1200 (98.36)	–
Caregiver employed	1209	574 (47.48)	635 (52.52)	11
Day care/schooling	1220	271 (22.21)	949 (77.79)	–
Caregiver receives support grant	1220	879 (72.05)	341 (27.95)	–

### HIV-infected cohort

Of 628 HIV-infected participants, both the HIV viral load and CD4^+^ T cell counts were available for the preceding 6-month period in 573 (91.24%). All HIV-infected participants received antiretroviral therapy at the time of enrollment. Moderate and severe immune suppression, as evidenced by CD4^+^ T cell counts percentages of 15–24% (moderate) and < 15% (severe) for age < 5 years and absolute values of 200–499 cells/µL (moderate) and < 200 cells/µL (severe) for children and adolescents 5 years and older, were documented in 118 of 578 (20.42%) results available, most being moderately immune suppressed (95, 80.51%). Virological suppression was documented in 367 of 574 (63.94%) participants, with HIV viremia > 1 000 copies/mL found in 113 (19.69%) participants.

**Figure 2:**
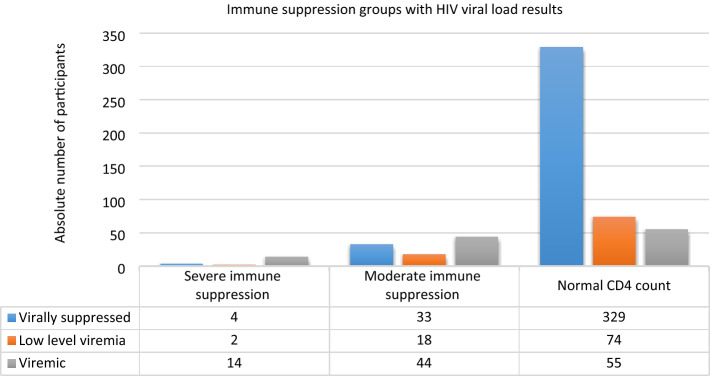
HIV-participant’s immune status and virological suppression. Immune suppression was classified as moderate (CD4^+^ T cell percentage 15–24% in < 5 yrs and absolute values 200–499/µL in ≥ 5 yrs of age) and severe (CD4^+^ T cell percentage < 15% in < 5 yrs and absolute values < 200/µL in ≥ 5 yrs of age). Viremia was classified as virally suppressed (< 50 copies/mL), low level (HIV viral load 50–999 copies/mL) and viremic (HIV viral load ≥ 1 000 copies/mL). * HIV-infected children and adolescent participants with available HIV viral load and CD4^+^ T cell counts in the past 6-month period.

Most HIV-infected children and adolescents (329, 71.83%) were both immunologically and virologically controlled or had only a low level of viremia < 1 000 copies/mL (74, 16.16%). Among the 20 (3.49) participants who were severely immune suppressed, 14/20 (70%) had a viremia of > 1 000 copies/mL (Fig. 2).

### HAV seroprevalence

**Figure 3:**
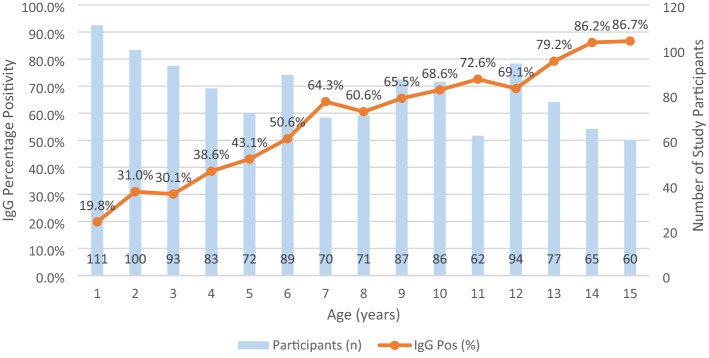
Hepatitis A seropositivity by age.

There were 672 (55.08%) participants who tested anti-HAV IgG positive and 32 (2.62%) anti-HAV IgM positive, all of which were also IgG positive. Increasing age was associated with an increased likelihood of testing IgG positive (OR 1.25; 95% CI 1.20–1.30, *p* < 0.001), with 19.8% of participants one year of age positive compared with 86.7% of participants 15 years of age (Fig. 3).

**Figure 4:**
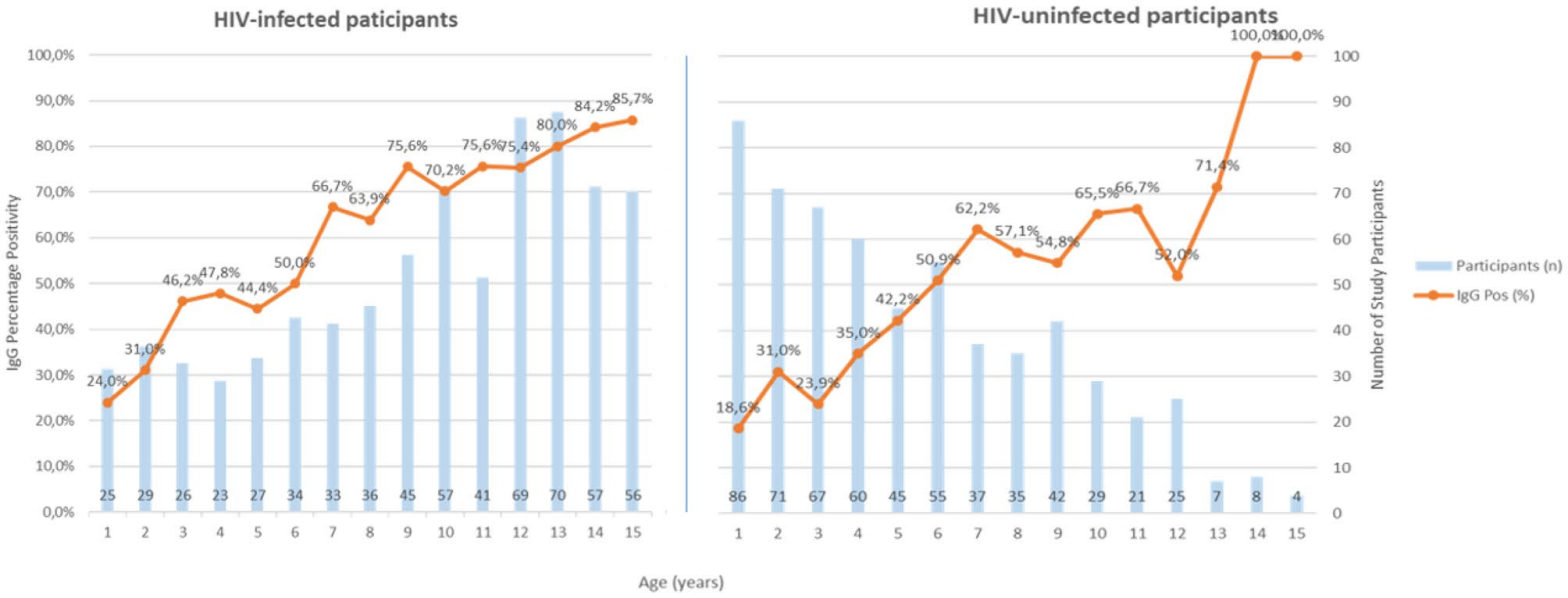
Hepatitis A seropositivity by age among HIV-infected and HIV-uninfected participants.

HAV IgG seropositivity increased with age among both HIV-uninfected and HIV-infected participants (Fig. 4).

In addition to older age, other variables associated with increased odds of testing IgG positive on univariate analysis were a positive HIV status of the participant, positive maternal HIV status during pregnancy, residing in an informal dwelling, use of a pit latrine at home, and having a primary caregiver who was employed. Variables associated with reduced odds of testing IgG positive were having a running water tap inside the home and attending daycare. However, on multivariate analysis, only age (OR 1.25; 95% CI 1.20–1.30, *p* < 0.001) and living in an informal dwelling (OR 2.27; 95% CI 1.41–3.67, *p* < 0.001) were statistically significant (Table 2). Further regression analysis restricted to participants < 12 yrs (age-groups with an n > 20), did not identify additional significant associations (Suppl. Table 1).

**Table 2:** Logistic Regression Analysis of Variables Associated with Hepatitis A Seropositivity.

Variable	Univariate			Multivariate		
	Odds ratio	95% CI	*P* value	Odds ratio	95% CI	*P* value
Age	1.24	1.21–1.28	< 0.001	1.25	1.20–1.30	< 0.001
HIV-status positive	2.84	2.26–3.58	< 0.001	1.11	0.78–1.57	0.57
Maternal HIV-status positive during pregnancy	2.04	1.63–2.56	< 0.001	1.22	0.88–1.68	0.24
Resides in an informal dwelling	2.09	1.62–2.73	< 0.001	2.27	1.41–3.67	< 0.001
Inside water tap	0.59	0.47–0.74	< 0.001	0.79	0.56–1.11	0.18
Pit latrine	2.06	1.56–2.73	< 0.001	1.07	0.67–1.72	0.77
Caregiver without schooling	0.94	0.39–2.27	0.89	–	–	–
Caregiver employed	1.33	1.06–1.66	0.012	1.09	0.85–1.41	0.50
Day care	0.31	0.24–0.41	< 0.001	0.96	0.68–1.35	0.80
Caregiver receives support grant	1.18	0.92–1.51	0.19	–	–	–

## Discussion

This seroprevalence surveillance study in healthy, HIV-infected and HIV-uninfected children and adolescents residing in a South African urban setting suggests that South Africa has an intermediate HAV seroprevalence. Seroprevalence rates < 90% by 10 years of age (68.6%) are reported. Increased age and informal dwellings are statistically associated with HAV seropositivity, while HIV status does not significantly influence HAV seropositivity rates when adjusted for the other variables.

Although HIV infection in children and adolescents and maternal HIV status showed a higher likelihood of the child/adolescent being anti-HAV IgG positive, HIV infection did not prove to be a significant indicator of HAV seropositivity in multivariate analysis. Compared to the South African national HIV data, our HIV-infected cohort demonstrated better (71.83%) immune and virological control. Virological testing yielded a 64% suppression rate, 16% low-level viremia, and 20% viremia. For South Africa, between July 2019 and June 2020, among all children and adolescents (aged 1–15 years) with an HIV viral load test, 49% were virally suppressed, 24% had low-level viremia, and 27% had viremia with VL > 1000 copies/ml^12^. Hence, our cohort demonstrated slightly better virological control than the national average. HIV status did not have a significant impact on HAV seroprevalence; therefore, we suggest that countries with a high HIV burden should consider similar prevention strategies in HIV-infected and HIV-uninfected children and adolescents.

Our data clearly illustrate that South Africa has now progressed to a country with an HAV intermediate endemicity rate, with HAV seroprevalence by 10 years of age only 68.6%, and > 90% seropositivity only reached beyond 15 years of age. Considerations for prevention strategies, such as HAV immunization, should include cost analyses. The cost-effectiveness of universal hepatitis A vaccination is well documented in other intermediate HAV endemicity regions, such as Argentina, Brazil, Chile, and Mexico. Patterson et al. reviewed patients presenting with hepatitis A at tertiary level hospitals Cape Town, South Africa, and calculated the total cost per hepatitis A hospitalization of $1935.41 for adult patients and $563.06 for pediatric patients. Furthermore, more than 1 in every 10 hepatitis A cases (13.3%) developed complicated hepatitis A or resulted in death^13^. Further to the high cost and morbidity/mortality, Bruckmann et al. showed that HAV is currently the leading cause of pediatric acute liver failure requiring transplantation in South Africa, with even further expenses and disease burden to the health sector^14^.

The study was limited by participant enrolment in a single urban centre. However, Kalafong Provincial Tertiary Hospital services a wide community of informal settlement households with poor access to clean water and sanitation. The General Household 2018 survey, released by Statistics South Africa^15^, showed that 13.1% of South African households were living in informal dwellings, 89.0% had access to an improved source of water, and 83.0% of households had improved sanitation. Our study participants were more frequently living in informal dwellings (26.7%), had less access to clean water sources inside the household (43.6%), and had less access to proper sanitation (77.9%) compared to the general South African average in 2018. This would likely have overestimated the HAV seroprevalence, making it even more evident that South Africa has reached an intermediate HAV endemicity.

## Conclusion

In conclusion, the intermediate prevalence of total anti-HAV among children and adolescents in South Africa suggests the country’s improvement of safe water and food supply, hygiene, and sanitation. However, it leaves older children and adults vulnerable to acute HAV infection, and there is a potential for outbreaks. The public health sector should consider raising public awareness through hygiene promotion and preventive measures such as vaccination.

## Supplementary Information

Supplementary Information.

## Data Availability

Raw data were generated at Kalafong Provincial Tertiary Hospital and the University of Pretoria. Derived data supporting the findings of this study are available from the corresponding author [NdP] on request.
