# Supplementary material for: Hepatitis A virus seroprevalence among children and adolescents in a high-burden HIV setting in urban South Africa
Source: Sci Rep. 2022 Nov 30;12:20688. doi: 10.1038/s41598-022-25064-x (PMC9712520; doi:10.1038/s41598-022-25064-x)
Supplement: Supplementary file 1 — Supplementary Information. [file 41598_2022_25064_MOESM1_ESM.docx]

**Suppl Table 1. Logistic Regression Analysis of Variables Associated with Hepatitis A Seropositivity Restricted to Participants Aged 1-12 years**

| **Variable** | **Univariate** | | | **Multivariate** | | |
| --- | --- | --- | --- | --- | --- | --- |
|  | **Odds**  **Ratio** | **95% CI** | **P-value** | **Odds Ratio** | **95% CI** | **P-value** |
| Age | 1.24 | 1.20 – 1.29 | <0.001 | 1.25 | 1.19 – 1.31 | <0.001 |
| HIV-status positive | 2.26 | 1.76 – 2.9 | <0.001 | 1.15 | 0.80 – 1.65 | 0.44 |
| Maternal HIV-status positive during pregnancy | 1.84 | 1.44 – 2.36 | <0.001 | 1.25 | 0.88 – 1.76 | 0.21 |
| Resides in an informal dwelling | 2.15 | 1.63 – 2.84 | <0.001 | 2.16 | 1.32 – 3.56 | 0.002 |
| Inside water tap | 0.56 | 0.43 – 0.71 | <0.001 | 0.76 | 0.53 – 1.10 | 0.15 |
| Pit latrine | 2.03 | 1.51 – 2.74 | <0.001 | 1.01 | 0.62 – 1.65 | 0.96 |
| Caregiver without schooling | 0.53 | 0.17 – 1.51 | 0.26 | - | - | - |
| Caregiver employed | 1.18 | 0.93 – 1.51 | 0.18 | 1.02 | 0.78 – 1.35 | 0.87 |
| Day care | 0.40 | 0.30 – 0.53 | <0.001 | 0.97 | 0.68 – 1.38 | 0.87 |
| Caregiver receives support grant | 1.15 | 0.88 – 1.50 | 0.31 | - | - | - |
